# Ectopic Splicing Disturbs the Function of *Xist* RNA to Establish the Stable Heterochromatin State

**DOI:** 10.3389/fcell.2021.751154

**Published:** 2021-10-14

**Authors:** Ruka Matsuura, Tatsuro Nakajima, Saya Ichihara, Takashi Sado

**Affiliations:** ^1^Department of Advanced Bioscience, Graduate School of Agriculture, Kindai University, Nara, Japan; ^2^Medical Institute of Bioregulation, Kyushu University, Fukuoka, Japan; ^3^Agricultural Technology and Innovation Research Institute, Kindai University, Nara, Japan

**Keywords:** *Xist* RNA, embryonic stem cells (ESCs), X chromosome inactivation, heterochromatin, gene silencing, RNA-FISH

## Abstract

Non-coding *Xist* RNA plays an essential role in X chromosome inactivation (XCI) in female mammals. It coats the X chromosome in cis and mediates the recruitment of many proteins involved in gene silencing and heterochromatinization. The molecular basis of how *Xist* RNA initiates chromosomal silencing and what proteins participate in this process has been extensively studied and elucidated. Its involvement in the establishment and maintenance of the X-inactivated state is, however, less understood. The *Xist*^*IVS*^ allele we previously reported is peculiar in that it can initiate XCI but fails to establish the inactive state that is stably maintained and, therefore, may provide an opportunity to explore how *Xist* RNA contributes to establish a robust heterochromatin state. Here we demonstrate that ectopic splicing taking place to produce *Xist*^*IVS*^ RNA disturbs its function to properly establish stable XCI state. This finding warrants the potential of *Xist*^*IVS*^ RNA to provide further insight into our understanding of how *Xist* RNA contributes to establish sustainable heterochromatin.

## Introduction

A subset of long non-coding RNA (lncRNA) associates with chromatin and regulates chromatin state. *Xist* RNA is one of the most extensively studied lncRNAs (Borsani et al., 1991; Brockdorff et al., 1991), which becomes upregulated from one of the two X chromosomes during the early development of female mammals (Kay et al., 1993) and induces X chromosome inactivation (XCI) to compensate for the dosage difference of X-linked genes between the sexes by associating with the X chromosome it originates from (Lyon, 1961; Clemson et al., 1996; Penny et al., 1996; Marahrens et al., 1997). In the mouse, XCI is imprinted in the extraembryonic lineages (Takagi and Sasaki, 1975), which contributes to the placenta and a part of the extraembryonic membranes, whereas it takes place in a random fashion to either X in the embryonic lineage, giving rise to whole tissues of the fetus (Monk and Harper, 1979). *Xist* RNA plays a pivotal role in both types of XCI by serving as a scaffold for recruiting many proteins involved in heterochromatin formation and maintenance.

Female mouse embryonic stem cells (ESCs) have been extensively used for the study of XCI as they carry two active X chromosomes, one of which undergoes chromosome-wide silencing with monoallelic upregulation of *Xist* and its association with the chromosome in cis upon induction of differentiation (Panning and Jaenisch, 1996). Functional domains of *Xist* RNA have been identified by studying ESCs introduced with a transgene expressing mutant *Xist* RNA with a deletion of various conserved domains among species. Deletion of the most proximal conserved repeat called the A-repeat not only totally compromises the silencing function of *Xist* RNA in the context of differentiating ESCs and developing embryos (Wutz et al., 2002; Sakata et al., 2017) but also abolishes an interaction of SPEN with *Xist* RNA (Chu et al., 2015; Moindrot et al., 2015; Monfort et al., 2015). A more recent study showed that the B-repeat interacts with hnRNPK to recruit PGF3/5-PRC1 to establish Polycomb-mediated chromosomal silencing (Pintacuda et al., 2017). Genetic screening and a more comprehensive proteomic analysis using ESCs have revealed many proteins that interact with *Xist* RNA (Chu et al., 2015; Moindrot et al., 2015; Pandya-Jones et al., 2020).

We previously reported a partial loss-of-function allele of *Xist*, *Xist^*IVS*^*, which initiates XCI in the extraembryonic tissues upon paternal transmission, but the XCI state initiated by *Xist*^*IVS*^ is not stable, resulting in derepression of X-inactivated genes in the extraembryonic tissues (Hoki et al., 2011). The embryos carrying the paternally derived *Xist*^*IVS*^ eventually die at the midgestation stage, at least, partly due to malfunction of the placenta. *Xist*^*IVS*^ was generated by introducing the 0.9-kb second intron of human γ-globin gene (IVS2) 0.9 kb downstream of the major transcription start site of *Xist* as a control allele for the analysis of antisense regulation by *Tsix* (Ohhata et al., 2008). When this allele is transcribed, the introduced IVS2 sequence is spliced out to produce essentially the same transcript as that transcribed from the wild-type allele except for the presence of an additional 16-base insertion derived from the targeting vector at the splicing junction. Although it was unknown whether or not the presence of such small insertion affects the function of *Xist* RNA, it was no doubt that the *Xist*^*IVS*^ allele is not fully functional. Unlike other mutant alleles of *Xist*, *Xist*^*IVS*^ is peculiar in that its RNA product can initiate appreciable levels of XCI in the extraembryonic tissues, which supports early postimplantation development, but fail to establish the stable XCI state. The *Xist*^*IVS*^ allele would, therefore, provide a unique opportunity to understand the molecular basis of how *Xist* RNA contributes to establishing the heritable robust heterochromatin, which allows stable maintenance of the X-inactivated state essential for embryonic development and healthy life after birth.

In this study, we addressed how *Xist*^*IVS*^ behaves in the embryonic tissue, in which random XCI takes place, and the impact of the presence of the 16-base insertion in *Xist* RNA on its function. The results demonstrate that the *Xist*^*IVS*^ allele is not upregulated in the embryonic tissue in contrast to that in the extraembryonic tissues, suggesting some difference in the mechanisms for upregulation of *Xist* between the embryonic and extraembryonic tissues. In addition, forced expression of *Xist*^*IVS*^ RNA from the newly generated *Xist^*CAGIVS*^* allele and another allele, *Xist*^*CAG*16*in*^, which produces the transcript containing the same 16-base insertion at the same position as *Xist*^*IVS*^ RNA without splicing, in differentiating ESCs demonstrates that the 16-base insertion per se does not affect the function of *Xist* RNA, indicating that splicing in the former transcript to remove the IVS2 sequence brings the qualitative difference between these transcripts. We discuss the potential of the *Xist*^*IVS*^ allele, which could facilitate our further understanding of how *Xist* RNA contributes to establish robust heterochromatin state of the inactive X chromosome.

## Materials and Methods

### Mice

*Xist*^*IVS*^ and *Xist*^1lox^ mice were described elsewhere (Sado et al., 2005; Hoki et al., 2011). The JF1 strain was maintained in-house and C57BL/6J strain purchased from Japan SLC, Inc. (Shizuoka, Japan). All mice were maintained and used in accordance with the Guidelines for the Care and Use of Laboratory Animals of Kindai University (KDAS-26-0006).

### Cells and Culture Condition

Embryonic stem cells used in this study were established from E3.5 blastocysts according to Ying et al. (2008). Blastocysts were cultured on four-well plates containing feeder cells with N2B27 medium supplemented with 1 μM of PD0325901 (Cayman Chemical, Ann Arbor, MI, United States) and 3 μM of CHIR99021 (Cayman) as well as 1,000 U/ml of LIF (Nacalai USA, San Diego, CA, United States). Outgrowths of undifferentiated cells were dissociated with TrypLE (Invitrogen, Carlsbad, CA, United States) and seeded on a four-well plate containing feeder cells. Many ESC colonies that appeared in several days were dissociated by TrypLE and seeded on a 35-mm dish containing feeder cells as passage 1 (P1). Blastocysts used for the establishment of ESCs homozygous for *Xist*^*IVS*^ were prepared from a cross between females heterozygous for *Xist*^*IVS*^ and males hemizygous for *Xist*^*IVS*^. Those used for the establishment of F1 hybrid female ESCs were prepared from a cross between JF1 (*Mus m. molossinus*) females and C57BL/6J (B6) males. JB4, one of the established ESCs, was adapted to grow on a gelatin-coated dish with conventional ESC medium containing 2i (DMEM containing 15% FBS, 1× non-essential amino acids, 1× penicillin/streptomycin, 0.1 mM β-mercaptoethanol, 1,000 U/ml of LIF, 1 μM of PD0325901, 3 μM of CHIR99021) for some passages. For induction of differentiation, ESCs were cultured with N2B27 medium on gelatin-coated dishes for up to 7 days.

### Construction of Targeting Vectors

For construction of a targeting vector for *Eif2s3x*, a 938-bp fragment present downstream of a termination codon of *Eif2s3x* was amplified by PCR using primers, Eif2s3x-F (5′-actctgtaga caaggctggc-3′) and Eif2s3x-R (5′-TTCTGTAGGGAGAATTGG CC-3′), on B6 genomic DNA and cloned into pBluescriptII-SK(+), in which the SpeI site present in MCS had been destroyed. This plasmid was linearized at the SpeI site present in the *Eif2s3x* fragment, and an IRES neo cassette was subsequently cloned in an appropriate orientation to generate p3′EifIRESneo. For construction of a targeting vector for *Hprt*, a 763-bp fragment containing an ATG start codon of *Hprt* was amplified by PCR using primers, gHprt-F1 (5′-agacgacagagggcctgggggctgc-3′) and gHprt-R1 (5′-ttgtagagctgggcctctcccagga-3′), on JF1 genomic DNA. This plasmid was used for inverse PCR using primers Hprt-invR(Spe) (5′-gggaaacttactagtCGGCAAAAAGC GGTCTGAGGAGGAAGC-3′) and Hprt-invF(Pst) (5′-gggaaact tctgcagCGACCCGCAGTCCCAGCGTCGTGgtga-3′), and the amplified fragment was circulized by self-ligation. The resultant plasmid was digested by *Pst*I and *Spe*I and used to clone a CAG-Zeo-pA cassette to produce pHprt_invF/R-Zeo.

For construction of a targeting vector to generate the *Xist*^*CAGIVS2lox*^ allele, a 0.9-kb IVS fragment amplified on genomic DNA carrying the *Xist*^*IVS*^ allele by PCR according to Ohhata et al. (2008) was cloned into a targeting vector, pCAG-CΔM20, which was used for replacing the endogenous *Xist* promoter with the CAG promoter (Amakawa et al., 2015), to generate pCAG-CΔM20-IVS#5. For construction of a targeting vector to generate the *Xist*^*CAG16in2lox*^ allele, an extra 16-base double-stranded DNA *Xist*^*IVS*^ RNA was introduced in pCAG-CΔM20 at the unique *Xho*I site to generate pCAG16in#3.

A 20-bp double-stranded DNA fragment corresponding to the respective specific guide RNA sequence was cloned into pX330 (Add genes) linearized by BbsI to generate pX330-EifgRNA1, pHprt-gRNA, and pX330-Xist(-20). sgRNA sequences are as follows: Eif2s3x (5′-ATTTATAGCTGCTACTAGTA-3′), Hprt (5′-TGACGGGACCGGTCGGCTCG-3′), and Xist (5′-GATCAGTTAAAGGCGTGCAA-3′).

### Establishment of Hybrid Female Embryonic Stem Cells Stably Maintaining Two X Chromosomes

The *Eif2s3x* locus, which is known to escape XCI, and the *Hprt* locus were selected for the site to introduce an IRESneo cassette and a CAGzeo-pA cassette, respectively. JB4 ESCs were used at P8 to introduce an IRESneo cassette at the *Eif2s3x* locus, and 5 × 10^5^ cells were transfected with 1 μg of pX330-EifgRNA1 and 1 μg of p3′EifIRESneo using FuGENE HD Transfection Reagent (Promega, Madison, WI, United States) and seeded on two gelatin-coated 60-mm dishes. Twenty-four hours later, selection was started with conventional ES medium containing 2i/LIF and 200 μg/ml of G418 for 8 days. Of the 40 colonies isolated, 12 turned out to harbor expected homologous recombination at one of the two *Eif2s3x* alleles. One of the 12 clones, termed JB4/EI7, which were confirmed to have an IRESneo cassette integrated on the X chromosome derived from B6, was used for targeting a CAGzeo-pA cassette at the *Hprt* locus on the X chromosome derived from JF1. Five hundred thousand cells were transfected with 1 μg of pHprt-gRNA and 1 μg of pHprt_invF/R-Zeo, as above. Selection was carried out using conventional ES medium containing 2i/LIF and 25 μg/ml of Zeocin for 10 days. Of the 40 colonies screened by PCR, one was found to harbor a correct homologous recombination at the *Hprt* locus on the X chromosome derived from JF1 and termed JB4/EI7HZ2. JB4/EI7HZ2 retained two X chromosomes in 99% of the population after 70 days of culture in the presence of G418 and Zeocin.

### Generation of Embryonic Stem Cells Carrying Either *Xist*^*CAGIVS2lox*^ or *Xist*^*CAG*16*in*2*lox*^

Five hundred thousand JB4/EI7HZ2 cells were transfected with 1 μg of pX330-Xist(-20) and 1 μg of either pCAG-CΔM20-IVS#5 or pCAG16in#3, as above. Selection was carried out using conventional ES medium containing 2i/LIF and 2 μg/ml of puromycin for 7–10 days. Twelve of 48 and 24 of 96 colonies isolated turned out to harbor *Xist*^*CAGIVS2lox*^ and *Xist*^*CAG16in2lox*^, respectively. To identify which of the B6 or JF1 alleles was targeted, a relevant region containing a single-nucleotide polymorphism (SNP) was amplified by PCR and analyzed by restriction digestion and sequencing. A floxed PacECFP-pA cassette was removed by transient expression of Cre recombinase to convert *Xist*^*CAGIVS2lox*^ and *Xist*^*CAG16in2lox*^ into *Xist*^*CAGIVS*^ and *Xist*^*CAG16in*^, respectively.

### RNA-FISH and Whole-Mount RNA-FISH

RNA-FISH was performed using cells fixed with either 4% paraformaldehyde (PFA) or Carnoy’s fixative as previously described (Sado et al., 2001). For PFA fixation, cells grown on a coverslip were fixed with 4% PFA and subsequently permeabilized with 0.5 % Triton X-100/0.5% BSA/PBS for 30 min and dehydrated through 70 and 100% ethanol. *Xist* and *Atrx* probes were prepared using pXist_SS12.9 and a BAC clone P23-260I15, respectively, as previously described (Sakata et al., 2017).

Whole-mount RNA-FISH was carried out according to Shiura et al. (2018). Briefly, embryos were first permeabilized in 0.1% Triton X-100/PBS for 10 s and fixed in 4% paraformaldehyde/PBS containing 0.1% Triton X-100 for 10 min. Following incubation in 2xSCC/0.05% Tween 20, 2xSSC/25% formamide/0.05% Tween 20, and 2xSSC/50% formamide/0.05% Tween 20 for 10 min each, the embryos were hybridized with *Xist* and *Atrx* probes overnight at 37°C, followed by washes for 5 min twice at 37°C in each of 2xSSC/50% formamide and 2xSSC/0.05% Tween 20 and subsequent counterstaining with Hoechst 33258.

For allele-specific RNA-FISH for *Xist*, probes were prepared according to Harris et al. (2019). Briefly, five B6- and JF1-specific oligonucleotides, the sequences of which were exactly the same as those designed by Harris et al. (2019) and contained an SNP between B6 and JF1, were labeled with Cy5 and Cy3, respectively, at their 3′-end. Five mask oligonucleotides complementary to the common 3′ part of the labeled oligonucleotides were also prepared. These Cy5-labeled B6-specific oligonucleotides, Cy3-labeled JF1-specific oligonucleotides, and mask oligonucleotides were included in hybridization buffer (2xSSC/10% dextran sulfate/2 mg/ml BSA/25% formamide) at a concentration of 5 μM for labeled and 10 μM for mask oligonucleotides. In the hybridization reaction, a Green-dUTP-labeled strand-specific probe for *Xist* prepared according to Shiura et al. (2018) was also included to validate the hybridization signal produced by Cy5- and Cy3-labeled oligonucleotides. Washing was carried out in 2xSSC/10% formamide for 30 min at 42°C twice and in 2xSSC for 5 min at room temperature.

### RT-PCR

cDNA synthesis was carried out using random hexamer on 1 μg of total RNA treated with DNaseI in the presence or absence of SuperScript III (Invitrogen). One-fiftieth of the reaction was used as a template for PCR using a primer set, R700P2, and F1063AS (Sado et al., 2005). The PCR product was subsequently digested with *Xho*I, whose restriction site is present in the fragment derived from wild-type *Xist* RNA but destroyed in that derived from *Xist*^*IVS*^ and *Xist*^16in^ (Hoki et al., 2011).

## Results

### The *Xist*^*IVS*^ Allele Did Not Become Upregulated in the Epiblast

**FIGURE 1:**
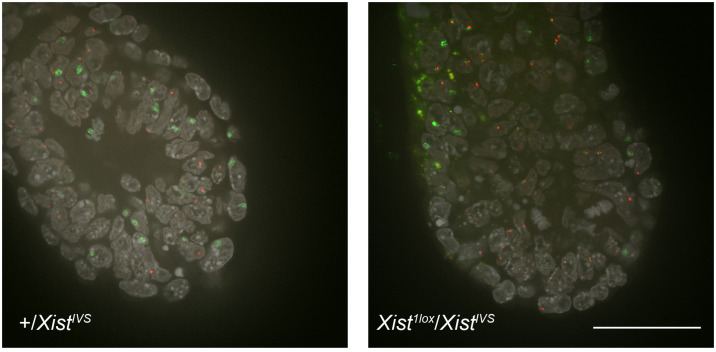
Whole-mount RNA-FISH in the distal part of E6.5 embryos. Expression of *Xist* (green) and *Atrx* (red) was examined in +/*Xist*^*IVS*^ and *Xist*^1lox^/*Xist*^*IVS*^ embryos recovered on E6.5. Six confocal sections of a 0.2-μm interval obtained by confocal microscopy were projected into a single image. epi, epiblast; ve, vesceral endoderm. Scale bar = 100 um.

To investigate the effect of *Xist*^*IVS*^ on XCI in the embryonic lineage, we established wild-type and *Xist*^*IVS*^/*Xist*^*IVS*^ ESCs from blastocysts obtained from a cross between *Xist*^*IVS*^/+ females and *Xist*^*IVS*^/Y males. They were induced to differentiate for 5 days and examined for *Xist* expression by RNA-FISH. While *Xist* was monoallelically upregulated in about 40% of wild-type cells, the majority of *Xist*^*IVS*^/*Xist*^*IVS*^ cells exhibited two pinpoint signals and none contained the *Xist* cloud (Supplementary Figure 1). Extension of differentiation did not change the situation. This raised a possibility that although the *Xist*^*IVS*^ allele induced XCI by coating the X chromosome in the extraembryonic lineages of the embryo, it was not upregulated in the embryonic lineage. To make a more direct assessment of this issue, we set out to examine whether or not the *Xist*^*IVS*^ allele became upregulated in the epiblast of the postimplantation embryo. We crossed females heterozygous for *Xist*^1lox^ with males hemizygous for *Xist*^*IVS*^, and embryos were recovered at embryonic day (E) 6.5 for whole-mount RNA-FISH. Since the *Xist*^1lox^ allele is functionally null and does not form the *Xist* cloud (Sado et al., 2005), RNA-FISH allowed us to evaluate the ability of the *Xist*^*IVS*^ allele to be upregulated in *Xist*^1lox^/*Xist*^*IVS*^ embryos. These embryos were morphologically reminiscent of previously reported *Xist*^*IVS*^/*Xist*^*IVS*^ embryos (Hoki et al., 2011), in which the epiblast is diminished, and therefore, they were differentiated from morphologically normal +/*Xist*^*IVS*^ female embryos, in which wild-type *Xist* is uniformly upregulated, and male embryos (*Xist*^1lox^/Y or +/Y) by visual inspection. Figure 1 shows the distal part of +/*Xist*^*IVS*^ and *Xist*^1lox^/*Xist*^*IVS*^ embryos examined at E6.5 for the expression of *Xist* and another X-linked *Atrx* gene by whole-mount RNA-FISH. In the +/*Xist*^*IVS*^ embryo, the *Xist* cloud was detected in the epiblast as well as the visceral endoderm layer surrounding the epiblast. *Atrx* was monoallelically expressed in these tissues, suggesting that one *Atrx* allele was silenced by XCI. In contrast, the *Xist* cloud was detected in the visceral endoderm but not in the epiblast of *Xist*^1lox^/*Xist*^*IVS*^ embryos. A pinpoint *Xist* signal detected was, however, often juxtaposed to a pinpoint signal of *Atrx*. Since even if RNA was transcribed from the *Xist*^1lox^ allele, it was truncated due to the insertion of an IRESEGFP-pA cassette in exon1 and would not be detected by the *Xist* probe used, and the pinpoint signals detected by the *Xist* probe should represent RNA transcribed from the *Xist*^*IVS*^ allele, suggesting a defect of the *Xist*^*IVS*^ allele to undergo upregulation. It was, therefore, likely that XCI was not induced in the epiblast of *Xist*^1lox^/*Xist*^*IVS*^ embryos, resulting in developmental failure of the epiblast. Taken together with the result in female ESCs homozygous for *Xist*^*IVS*^, we concluded that the *Xist*^*IVS*^ allele was defective in upregulating its transcription in the epiblast lineage. This contrasts with the fact that the *Xist*^*IVS*^ allele is upregulated and capable of inducing XCI in the extraembryonic lineages and suggests some differences in the mechanism of *Xist* upregulation between the embryonic and extraembryonic lineages.

### Forced Expression of *Xist*^*IVS*^ RNA Compromised XCI in a Fraction of Differentiating Embryonic Stem Cells

To circumvent the defect of the *Xist*^*IVS*^ allele to be upregulated, we attempted to replace the endogenous *Xist* promoter with the CAG promoter (Niwa et al., 1991) in F1 hybrid female ESCs between JF1 and B6 by homologous recombination with the aid of CRIPSR/Cas9 genome editing (Supplementary Figure 2). ESCs used here carried the neomycin resistance gene on the X chromosome derived from B6 (X^B6^) and the Zeocin resistance gene on the X derived from JF1 (X^JF1^) and, therefore, stably maintained both X chromosomes in the presence of G418 and Zeocin in culture medium (see *Materials and methods*). We isolated several lines harboring the *Xist*^*CAGIVS*2*lox*^ allele on either X chromosome and chose two lines, one targeted on the X^B6^ (IVS-2L-B47) and the other on the X^JF1^ (IVS-2L-J19), for further analyses. A floxed PacECFPpA cassette was removed by transient expression of Cre recombinase in IVS-2L-B47 and IVS-2L-J19 lines to obtain IVS-B47#24 and IVS-J19#3, respectively, in which the *Xist*^*CAGIVS2lox*^ allele was converted into the *Xist*^*CAGIVS*^ allele.

**FIGURE 2:**
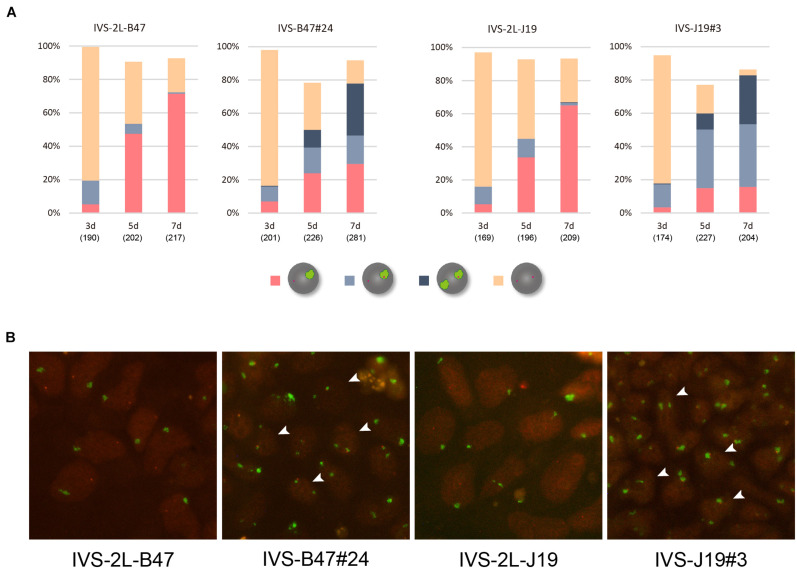
RNA-FISH in differentiating ESCs heterozygous for either *Xist*^*CAGIVS2lox*^ or *Xist^*CAGIVS*^*. **(A)** Expression of *Xist* and *Atrx* was examined in respective differentiating ESCs for day 3 (d3) up to day 7 (d7). IVS-2L-B47 and IVS-B47#24 carry the targeted *Xist* allele on the X^B6^; IVS-2L-J19 and IVS-J19#3 on the X^JF1^. The number of nuclei examined is indicated in parentheses under each day point. **(B)** Representative images of RNA-FISH in each ESC line on d7. *Xist* in green and *Atrx* in red. Arrowhead indicates some of the nuclei containing two *Xist* clouds, one of which juxtaposes an *Atrx* signal.

These ESC lines thus generated carrying either *Xist*^*CAGIVS2lox*^ (IVS-2L-B47 and IVS-2L-J19) or *Xist*^*CAGIVS*^ (IVS-B47#24 and IVS-J19#3) were allowed to differentiate for up to 7 days in N2B27 medium without G418 and Zeocin, and expression of *Xist* and X-linked *Atrx* was examined by RNA-FISH. Given our previous studies of the targeted *Xist* alleles generated in a similar scheme, *Xist*^*CAG2L*^ and *Xist^*CAG*Δ5^′^–2*L*^* (Amakawa et al., 2015; Sakata et al., 2017), it was reasonable to expect that the *Xist*^*CAGIVS2L*^ allele would behave as a functionally null allele and the other wild-type allele would be upregulated upon differentiation. In IVS-2L-B47 and IVS-2L-J19 cells, the proportion of cells containing the *Xist* cloud increased over time to reach 65–70% of the population at day 7 (d7) (Figures 2A,B). Expression of *Atrx* was detected as a single pinpoint signal, which did not overlap with the *Xist* cloud, suggesting that *Atrx* on the *Xist* RNA-coated X was silenced. In IVS-B47#24 and IVS-J19#3 cells, although the *Xist*^*CAGIVS*^ allele was driven by the CAG promoter, it was not upregulated prior to differentiation as previously reported (Amakawa et al., 2015). Following differentiation, although gradual upregulation of *Xist* was similarly observed in IVS-B47#24 and IVS-J19#3 cells over time, a large fraction of cells with a single *Xist* cloud expressed *Atrx* biallelically with expression of one allele overlapping with the *Xist* cloud at d5 and d7. In the remaining fraction of cells with a single *Xist* cloud, *Atrx* was monoallelically expressed, and its signal did not overlap with the *Xist* cloud. In addition, there was a gradual increase of cells that contained two *Xist* clouds, one of which overlapped with an *Atrx* signal. Such cells represented ∼30% of the population by d7. These results suggested that one of the two *Xist* clouds detected in a fraction of differentiating IVS-B47#24 and IVS-J19#3 cells allowed misexpression of *Atrx* and, therefore, was defective in silencing the X chromosome.

### Kinetics of Allelic Expression of *Xist* in Differentiating Embryonic Stem Cells Heterozygous for *Xist*^*CAGIVS*^

To identify the allelic origin of *Xist* RNA forming the *Xist* cloud in differentiating female ESCs, we employed allele-specific RNA-FISH using labeled B6- and JF1-specific oligo probes that differentially hybridized to *Xist* RNA derived from the B6 and JF allele, respectively (Harris et al., 2019). This confirmed that only the wild-type *Xist* allele expressed from the X^JF1^ and X^B6^ formed the cloud in IVS-2L-B47 and IVS-2L-J19 cells, respectively, at day 7 of differentiation.

**FIGURE 3:**
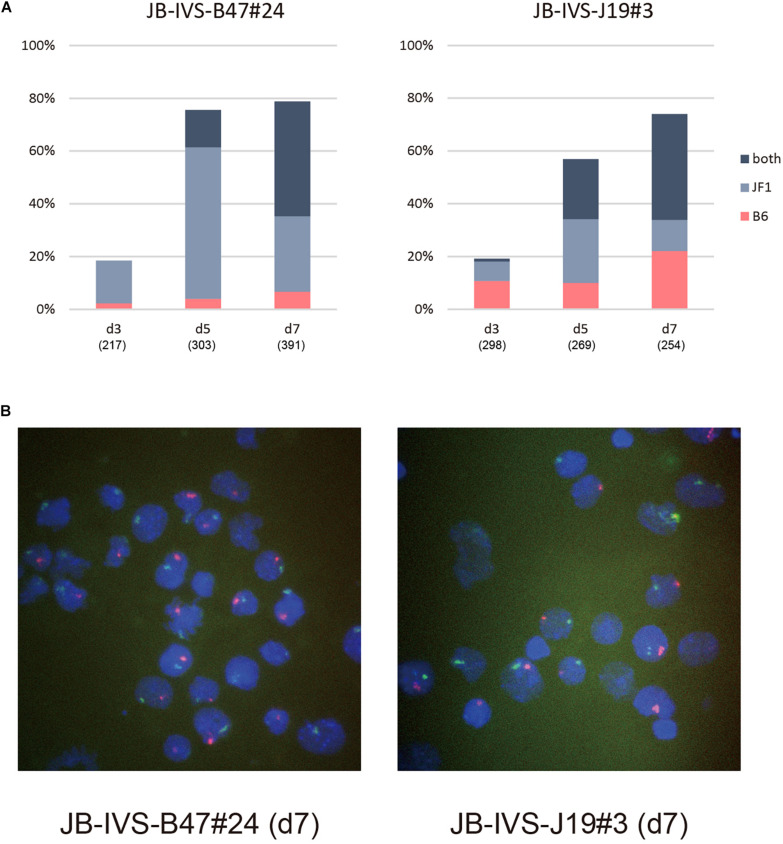
Allele-specific RNA-FISH for *Xist* in IVS-B47#24 and IVS-J19#3. **(A)** Proportion of nuclei harboring the *Xist* cloud of the X^B6^ or X^JF1^ in origin or both in respective differentiating ESCs for d3 up to d7. IVS-B47#24 carries *Xist*^*CAGIVS*^ on the X^B6^ and IVS-J19#3 on the X^JF1^. **(B)** Representative images of allele-specific RNA-FISH for *Xist* in each ESC line on d7. *Xist* RNA originated from the X^B6^ and X^JF1^ is shown in red and green, respectively.

Subsequently performed allele-specific *Xist* RNA-FISH revealed that *Xist* RNA forming the cloud in IVS-B47#24 cells was unexpectedly biased toward the RNA occurring from the wild-type allele on the X^JF1^ rather than the one from the *Xist*^*CAGIVS*^ allele on the X^B6^ on d3 (Figures 3A,B). Although this bias was more pronounced on d5, two *Xist* clouds detected in a subset of cells were indeed originated from the wild-type and *Xist*^*CAGIVS*^ alleles on the X^B6^ and X^JF1^, respectively. The population of cells containing two *Xist* clouds increased from d5 to d7 with a decrease in the proportion of cells containing the single cloud of wild-type *Xist* on the X^JF1^. This suggested that the *Xist*^*CAGIVS*^ allele became upregulated in a subset of cells, which had undergone differentiation and initiated XCI *via* upregulation of wild-type *Xist* on the X^JF1^, in IVS-B47#24 cells.

The kinetics of allelic expression of *Xist* in IVS-J19#3 cells was different. Cells that had initiated XCI by d3 seemed to have selected either the wild-type allele on the X^B6^ or the *Xist*^*CAGIVS*^ allele on the X^JF1^ for upregulation in a random fashion (Figures 3A,B). Those that upregulated the *Xist*^*CAGIVS*^ allele, however, had dominated in the population carrying the single *Xist* cloud by d5. As was the case in IVS-B47#24 cells, the proportion of these cells subsequently decreased from d5 to d7 with an increase of those containing two *Xist* clouds, which were derived from the wild-type and *Xist*^*CAGIVS*^ allele on the X^B6^ and X^JF1^, respectively. This suggested that the wild-type *Xist* became upregulated in a subset of cells, which had upregulated the *Xist*^*CAGIVS*^ first during differentiation.

Although we expected that the *Xist*^*CAGIVS*^ allele driven by the CAG promoter was preferentially upregulated upon differentiation, allele-specific RNA-FISH suggested that it was not necessarily the case and either allele could be chosen for upregulation when XCI initiated. Although two *Xist* clouds were subsequently formed in both IVS-B47#24 and IVS-J19#3 cells, the kinetics to become the two cloud states appeared different. Nonetheless, allele-specific RNA-FISH confirmed that the two *Xist* clouds were indeed formed by wild-type *Xist* RNA and *Xist*^*IVS*^ RNA derived from the *Xist*^*CAGIVS*^ allele. It was most likely that *Xist*^*IVS*^ RNA was the one forming the cloud defective in *Atrx* silencing, and therefore, it was functionally compromised.

### *Xist*^*IVS*^ RNA Was Defective Because It Underwent Splicing

**FIGURE 4:**
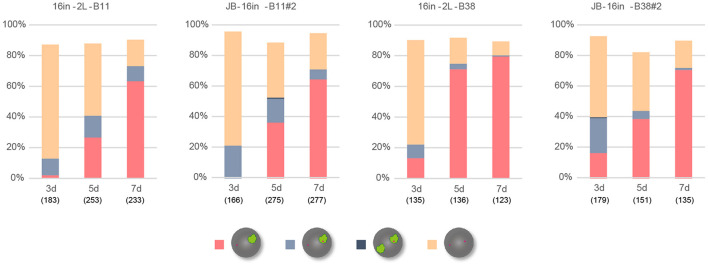
RNA-FISH in differentiating ESCs heterozygous for either *Xist*^*CAG16in2lox*^ or *Xist*^*CAG16in*^. Expression of *Xist* and *Atrx* was examined in respective differentiating ESCs for day 3 (d3) up to day 7 (d7). Each cell line examined (16in-2L-B11, 16in-B11#2, 16in-2L-B38, and 16in-B38#2) carried the targeted *Xist* allele on the X^B6^. The number of nuclei examined is indicated in parentheses under each day point.

*Xist*^*IVS*^ RNA inevitably contains an additional 16-base insertion derived from the targeting vector after splicing of the IVS2 sequence (Ohhata et al., 2008). To address the impact of the presence of the 16-base insertion on the function of *Xist* RNA, we generated another female ESCs harboring the *Xist*^*CAG16in*^ allele, which produced *Xist*^16in^ RNA containing exactly the same 16-base insertion at the same position without splicing under the control of the CAG promoter (Supplementary Figure 3). The ESC lines thus generated, 16in-B11#2 and 16in-B38#2, which carried the targeted allele on the X^B6^ (those carrying the targeted allele on the X^JF1^ were not recovered), and their parental line containing a floxed PacECFPpA cassette, 16in-2L-B11 and 16in-2L-B38, respectively, were allowed to differentiate in N2B27 medium and examined for *Xist* and *Atrx* expression by RNA-FISH. In all cases, the proportion of cells containing the *Xist* cloud gradually increased over time (Figure 4) and *Xist* RNA forming a single cloud essentially silenced *Atrx* as few overlaps were observed. This suggested that *Xist*^16in^ RNA was capable of inducing and establishing the stably silenced state of the X chromosome. In contrast to IVS-B47#24 and IVS-J19#3 cells, only a minor population of the cells showed two *Xist* clouds in 16in-B11#2 and 16in-B38#2.

**FIGURE 5:**
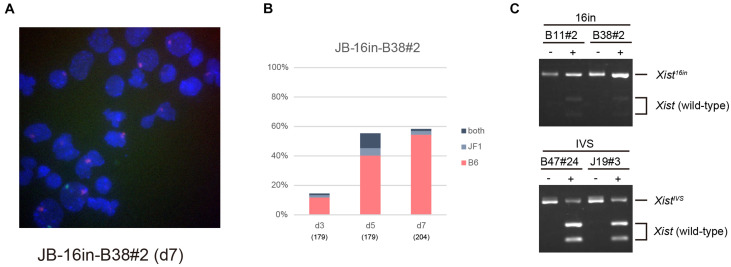
*Xist*^16in^ RNA induces stable XCI. **(A)** Representative images of allele-specific RNA-FISH for *Xist* in 16in-B38#2 differentiated for 7 days. *Xist* RNA originated from the X^B6^ and X^JF1^ is shown in red and green, respectively. **(B)** Proportion of nuclei harboring the *Xist* cloud of the X^B6^ or X^JF1^ in origin or both in differentiating 16in-B38#2 ESCs for d3 up to d7. **(C)** Expression of *Xist*^16in^ and *Xist*^*IVS*^ RNA in differentiating ESCs heterozygous for the *Xist*^16in^ and *Xist*^*CAGIVS*^ allele, respectively. The unique *Xho*I site present in the amplified fragment derived from wild-type *Xist* RNA was destroyed by the presence of the 16-base insertion in the amplified fragment derived from *Xist*^16in^ and *Xist*^*IVS*^ RNA. The presence of an undigested band by *Xho*I indicates the expression of *Xist*^16in^ and *Xist*^*IVS*^ RNA. (–) and (+) indicate undigested and digested, respectively.

Allele-specific RNA-FISH for *Xist* revealed that the RNA forming the majority of the cloud derived from X^B6^, on which the *Xist*^16in^ allele had been introduced, in 16in-B38#2 on d7 (Figures 5A,B). RT-PCR and subsequent restriction digestion of the amplified fragment further confirmed the expression of *Xist*^16in^ RNA in 16in-B11#2 and 16in-B38#2 (Figure 5C). The same analysis also demonstrated the production of not only the expected spliced product from the *Xist^*CAGIVS*^* allele but also wild-type *Xist* RNA in IVS-B47#24 and IVS-J19#3 (Figure 5C), consistent with allele-specific RNA-FISH for *Xist*, shown in Figure 3. These results indicated that *Xist*^16in^ RNA was the one that formed the cloud stably silencing *Atrx* in 16in-B38#2 cells. *Xist*^16in^ RNA was therefore competent to induce stable XCI even though it contains exactly the same 16-base insertion at the same position as *Xist*^*IVS*^ RNA. It is, therefore, reasonable to conclude that the presence of the 16-base insertion per se does not compromise the function of *Xist* RNA, and it is splicing that deteriorates the function of the RNA expressed from the *Xist*^*CAGIVS*^ allele.

## Discussion

### *Xist*^*IVS*^ Was Not Upregulated in the Embryonic Tissues

We previously reported that although *Xist*^*IVS*^ RNA expressed in the extraembryonic tissues could not establish a stable XCI state, it could support the early postimplantation development by inducing appreciable levels of XCI (Hoki et al., 2011). We also described that the *Xist* cloud was detected in 70% of the nuclei but absent in the remaining 30% in the distal part of E7.5 *Xist*^*IVS*^/*Xist*^*IVS*^ embryos dissociated by treatment with lactic acid. This led us to speculate that although the *Xist*^*IVS*^ allele was initially monoallelically upregulated, the transcripts coating one of the two Xs was lost over time in the embryonic tissue. However, the relative abundance of the epiblast cells in the distal part of an E7.5 embryo, which consists of the epiblast and visceral endoderm, would be much lower in *Xist*^*IVS*^/*Xist*^*IVS*^ embryos characterized by the small epiblast that fails to expand than in the morphologically normal wild-type embryos. Inevitable contamination of a relatively large proportion of the visceral endoderm in the mutant embryos might have led us to misinterpretation. In this study, however, whole-mount RNA-FISH using E6.5 compound heterozygotes, *Xist*^1lox^/*Xist*^*IVS*^, unequivocally demonstrated that the upregulation of the *Xist*^*IVS*^ allele was confined only to the visceral endoderm and no *Xist* cloud was formed in the epiblast in the distal part of the embryo. This finding together with RNA-FISH analysis of ESCs homozygous for *Xist*^*IVS*^ indicated that *Xist*^*IVS*^ was not upregulated in the embryonic tissue. The differential behavior of the *Xist*^*IVS*^ allele between the embryonic and extraembryonic tissues is most probably related to the mode of XCI, that is, imprinted or random. While in the tissues that undergo imprinted XCI, *Tsix*, an antisense RNA of *Xist*, is expressed on the maternal X but not on the paternal X, it is biallelically expressed prior to upregulation of *Xist* in undifferentiated epiblast cells and ESCs, which undergo random XCI as cells differentiate (Lee, 2000; Sado et al., 2001). Given that *Tsix* negatively regulates *Xist* through modification of chromatin structure (Navarro et al., 2005; Sado et al., 2005), it is possible that the presence or absence of *Tsix* RNA or its transcription affects the potential of the *Xist*^*IVS*^ allele to be upregulated in response to cellular differentiation. *Tsix* expression is downregulated on one of the two Xs in the epiblast or ESCs during differentiation, and *Xist* becomes upregulated on that X to induce XCI (Lee et al., 1999). A series of these events may be compromised on the X chromosome carrying the *Xist*^*IVS*^ allele in the epiblast cells or ESCs. In the extraembryonic tissues, on the other hand, since *Tsix* is imprinted not to be expressed on the paternal X, there would not be any influence of *Tsix* on the paternal *Xist*^*IVS*^ allele, allowing its upregulation.

### Splicing Deteriorates the Function of *Xist*^*IVS*^ RNA

We expected that when female ESCs heterozygous for *Xist*^*CAGIVS*^ were allowed to differentiate, the *Xist*^*CAGIVS*^ allele would become preferentially upregulated and its transcript, *Xist*^*IVS*^ RNA, would coat the X chromosome. Allele-specific RNA-FISH, however, revealed that this was not the case and either the wild-type or the *Xist*^*CAGIVS*^ allele could be monoallelically upregulated at the onset of XCI. Although we had to admit that the *Xist*^*CAGIVS*^ allele behaved a little different way from the one that we expected during the early phase of differentiation, still we could express the *Xist*^*IVS*^ RNA in differentiating ESCs as a result of the formation of two *Xist* clouds in both cell lines, IVS-B47#24 and IVS-J19#3. We speculate that in IVS-B47#24, although the wild-type *Xist* allele on the X^JF1^ was initially preferentially upregulated upon initiation of XCI, the subsequent differentiated state of the cells allowed them to activate the CAG promoter, which barely drives transcription at the *Xist* locus in undifferentiated state, to express *Xist*^*IVS*^ RNA from the *Xist*^*CAGIVS*^ allele. In IVS-J19#3, on the other hand, one of either allele of *Xist* was selected for upregulation at the onset of XCI; the wild-type *Xist* allele appeared secondarily activated in those that upregulated the *Xist^*CAGIVS*^* allele first to compensate for the insufficient XCI initiated by *Xist*^*IVS*^ RNA. It is likely that those that upregulated the wild-type *Xist* first in the population of IVS-J19#3 formed two *Xist* clouds in the same manner as IVS-B47#24. Whatever the reason for the formation of two *Xist* clouds is, upregulation of the *Xist^*CAGIVS*^* allele allowed us to evaluate the function of *Xist*^*IVS*^ RNA in the embryonic lineage. Since one of the two *Xist* clouds seemed to be defective in silencing *Atrx*, it was most likely that *Xist*^*IVS*^ RNA produced from the *Xist*^*CAGIVS*^ allele was dysfunctional and failed to establish the proper XCI state. In contrast, *Xist*^16in^ RNA containing the same 16-base insertion at the same position as *Xist*^*IVS*^ RNA was indistinguishable from wild-type *Xist* RNA in terms of the kinetics of *Atrx* silencing. This strongly suggests that it is not the 16-base insertion per se that compromises the function of *Xist*^*IVS*^ RNA. The difference between *Xist*^*IVS*^ RNA and *Xist*^16in^ RNA is whether or not the RNA undergoes splicing to remove the IVS2 sequence introduced 0.9 kb downstream of the major transcription start site of *Xist*. When an intron is removed and two exons are connected by splicing, many splicing-related proteins such as an exon junction complex (EJC) bind in the vicinity of the exon-exon junction. It is, therefore, reasonable to expect that such proteins would bind to the processed *Xist*^*IVS*^ RNA but not to *Xist*^16in^ RNA. The IVS2 sequence was located about 0.2 kb downstream of the A-repeat, which is essential for the silencing function of *Xist* RNA (Wutz et al., 2002) and mediates binding of some important proteins required for XCI such as SPEN (Chu et al., 2015; McHugh et al., 2015; Moindrot et al., 2015; Monfort et al., 2015) and RBM15 (Patil et al., 2016). We are tempted to speculate that binding of these factors to *Xist* RNA is disturbed by the presence of EJC and other factors brought on *Xist*^*IVS*^ RNA as a result of splicing to remove the IVS2 sequence. It would be therefore particularly interesting to compare the proteins assembled on *Xist*^*IVS*^ RNA with those on the wild-type *Xist* RNA. We cannot, however, exclude the possibilities that the inefficient silencing associated with *Xist*^*IVS*^ RNA could result from the efficiency of the expected splicing event on the premature *Xist*^*IVS*^ RNA or the difference of the overall levels of wild-type *Xist*, *Xist*^*IVS*^, and *Xist*^16in^ RNA. The fact that differentiating ESCs carrying the *Xist*^*CAGIVS*^ allele give rise to the populations with biallelic expression of *Xist* (wild-type and *Xist*^*IVS*^) as well as monoallelic expression of either allele makes it difficult to compare the quantity of the RNA produced from the respective wild-type and *Xist*^*CAGIVS*^ allele. To circumvent this problem, we are currently attempting to derive a unique cell population with monoallelic expression of either allele or that with biallelic expression of both by inducing differentiation of ESCs into neural stem cells and subsequent cloning.

Since most of the *Xist* mutant allele so far generated compromise the initiation process of XCI, *Xist*^*IVS*^ RNA is peculiar in that it can initiate XCI but fails to maintain the XCI state. This is most probably due to the failure of the establishment of robust heterochromatin. It is likely that proteins recruited by *Xist* RNA contribute to not only the initiation of XCI but also the establishment of a sustainable heterochromatin state. *Xist*^*IVS*^ RNA would provide a unique opportunity to explore such factors involved in the latter process and how *Xist* participates in the establishment of the chromatin state required for the stable maintenance of the X-inactivated state.

## Data Availability Statement

The original contributions presented in the study are included in the article/Supplementary Material, further inquiries can be directed to the corresponding author/s.

## Ethics Statement

The animal study was reviewed and approved by the committee for the Care and Use of Laboratory Animals of Kindai University.

## Author Contributions

TS and TN designed the study. RM, TN, SI, and TS performed the experiments and analyzed the data. TS wrote the manuscript. All authors contributed to the article and approved the submitted version.

## Conflict of Interest

The authors declare that the research was conducted in the absence of any commercial or financial relationships that could be construed as a potential conflict of interest.

## Publisher’s Note

All claims expressed in this article are solely those of the authors and do not necessarily represent those of their affiliated organizations, or those of the publisher, the editors and the reviewers. Any product that may be evaluated in this article, or claim that may be made by its manufacturer, is not guaranteed or endorsed by the publisher.
