# Supplementary material for: Ectopic Splicing Disturbs the Function of Xist RNA to Establish the Stable Heterochromatin State
Source: Front Cell Dev Biol. 2021 Oct 14;9:751154. doi: 10.3389/fcell.2021.751154 (PMC8551810; doi:10.3389/fcell.2021.751154)
Supplement: Supplementary file 1 [file Data_Sheet_1.PDF]

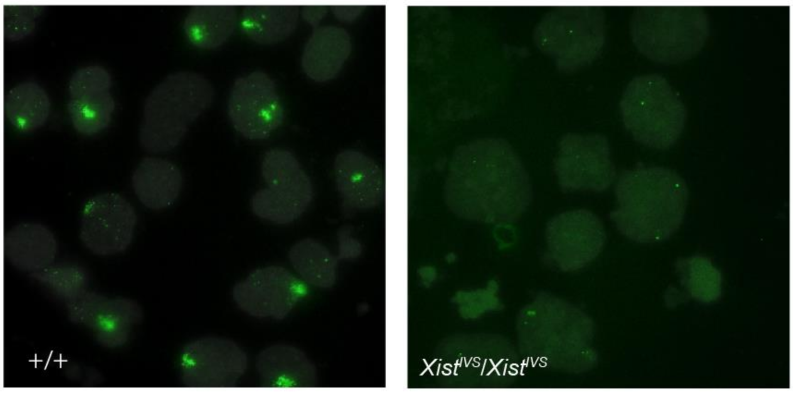

Supplementary Figure S1. RNA-FISH for *Xist* in differentiating ESCs.

RNA-FISH for *Xist* in differentiating wild-type female ESCs (+/+) and those homozygous for *Xist*^{IVS}/*Xist*^{IVS}. The *Xist* was never upregulated in *Xist*^{IVS}/*Xist*^{IVS} cells.

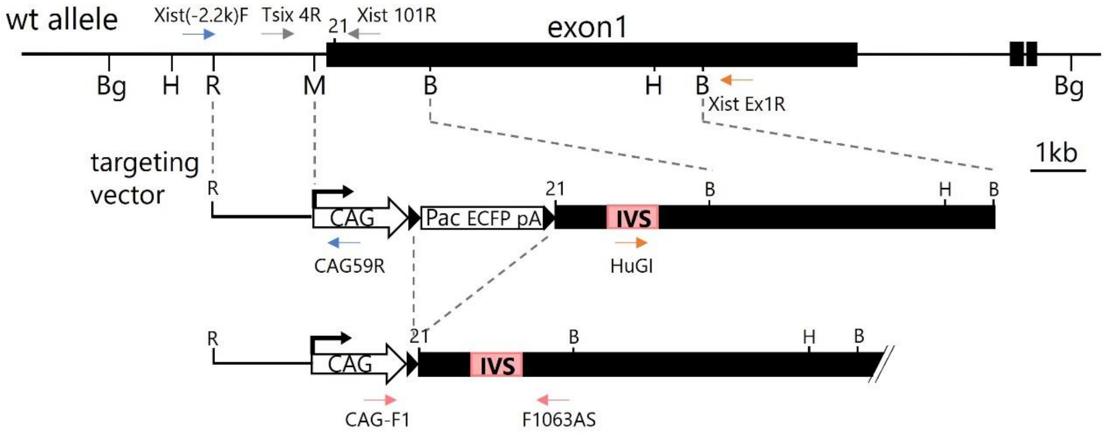

Supplementary Figure S2. Targeting scheme to introduce the *Xist*^{IVS} allele driven by the CAG promoter at the endogenous *Xist* locus.

Homologous recombination was facilitated by CRISPR/Cas9 mediated genome editing. An sgRNA was designed 20-nt upstream of the major transcription start site of Xist, the sequence of which was lost when the homologous recombination was successfully takes place between the genome and the targeting vectors. Successful targeting event was confirmed by PCR using a series of primers shown with arrows.

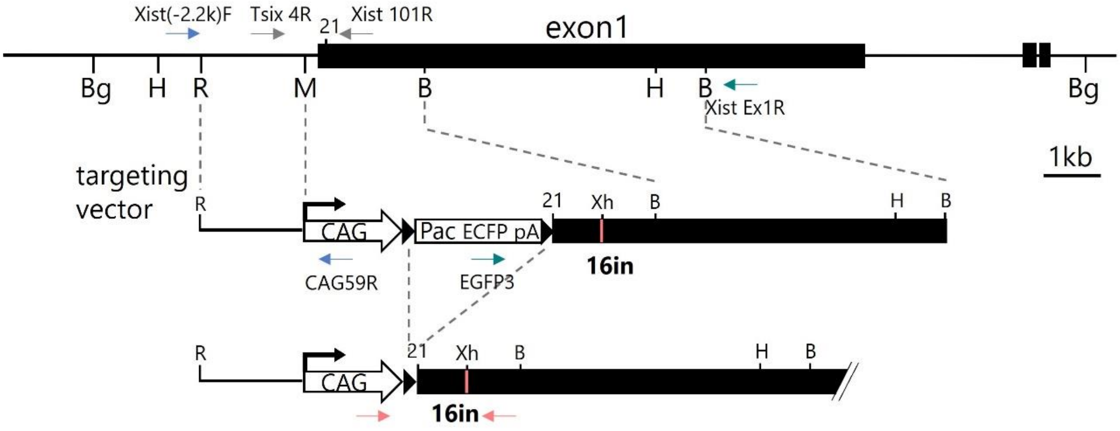

Supplementary Figure S3. Targeting scheme to introduce the *Xist*^{CAG16in} allele driven by the CAG promoter at the endogenous *Xist* locus.

Gene targeting was carried out in essentially the same way as the one for the generation of the *Xist*^{CAGIVS2lox} allele. Successful targeting event was confirmed by PCR using a series of primers shown with arrows.
